# Managing protected health information in distributed research network environments: automated review to facilitate collaboration

**DOI:** 10.1186/1472-6947-13-39

**Published:** 2013-03-22

**Authors:** Christine E Bredfeldt, Amy Butani, Sandhyasree Padmanabhan, Paul Hitz, Roy Pardee

**Affiliations:** 1 Mid-Atlantic Permanente Research Institute, Kaiser Permanente in the Mid-Atlantic States, Rockville, MD, USA; 2 HealthPartners Institute for Education and Research, Bloomington, MN, USA; 3 C-V Sight, Shrewsbury, MA, USA; 4 Essentia Institute of Rural Health, Duluth, MN, USA; 5 Group Health Research Institute, Seattle, WA, USA

**Keywords:** HIPAA, Protected health information, Distributed research, De-identification

## Abstract

**Background:**

Multi-site health sciences research is becoming more common, as it enables investigation of rare outcomes and diseases and new healthcare innovations. Multi-site research usually involves the transfer of large amounts of research data between collaborators, which increases the potential for accidental disclosures of protected health information (PHI). Standard protocols for preventing release of PHI are extremely vulnerable to human error, particularly when the shared data sets are large.

**Methods:**

To address this problem, we developed an automated program (SAS macro) to identify possible PHI in research data before it is transferred between research sites. The macro reviews all data in a designated directory to identify suspicious variable names and data patterns. The macro looks for variables that may contain personal identifiers such as medical record numbers and social security numbers. In addition, the macro identifies dates and numbers that may identify people who belong to small groups, who may be identifiable even in the absences of traditional identifiers.

**Results:**

Evaluation of the macro on 100 sample research data sets indicated a recall of 0.98 and precision of 0.81.

**Conclusions:**

When implemented consistently, the macro has the potential to streamline the PHI review process and significantly reduce accidental PHI disclosures.

## Background

Studying rare outcomes, new interventions, and diverse populations frequently requires collaborations across multiple healthcare institutions. As the capacity to exchange health research data grows through the development of distributed research networks, healthcare research collaboratories, and computing grids, the amount of new public health research involving partnerships across academic institutions, healthcare delivery systems, insurance providers and pharmaceutical companies is also growing [1]. Existing large-scale multi-site research and public health collaborations include HMO Research Network (HMORN) [2] based projects such as the Cancer and Cardiovascular Research Networks, the Vaccine Safety Datalink [3], and the Centers for Education and Research on Therapeutics [4], the FDA Sentinel project [5] and the Scalable PArtnering Network (SPAN) [6], among others. These collaborations often require the release of aggregated patient data or fully or partially de-identified patient-level information from participating institutions to the lead research site. Occasionally fully-identifiable patient information is required, subject to Institutional Review Board (IRB) approval and appropriate Data Use Agreements (DUAs).

In accordance with the Privacy and Security Regulations of the Health Insurance Portability and Accountability Act of 1996 (HIPAA), collaborating institutions work closely with their IRB to ensure that protected health information (PHI) used in research meets the “minimum necessary” requirements and has all appropriate safeguards [7]. As public health research collaborations grow more common, the potential for accidental disclosure of PHI also grows. Over the last several years, the authors have been aware of several accidental disclosures including temporary data that were accidentally released to the lead research site, multi-site extraction programs that failed to completely replace patient identifiers with de-identified study ids, and programmers who forgot to remove one or more of the 18 patient identifiers described by the Privacy Rule from the final data sets. Given the tight deadlines and complex data sets often required by multi-site research, accidental inclusion of PHI in research data sets is a real concern. Methods that make it easier to identify PHI and determine whether it is authorized for a given research project could significantly reduce accidental unauthorized PHI disclosures.

Effective PHI management requires efforts at multiple levels including national and organizational policy interpretation, access control, and control over data release. Ontology-based models have been developed to implement national and organizational policy as rule-based systems that control data access based on a complex interrelationship between the data user, the patient, and the purpose of the data use [8]. These systems control who has access to the data using metadata attached to the data elements. At the data release level, the goal is generally to release the least amount of personal data necessary to achieve the scientific objective. To that end, there have been several efforts to develop methods to scrub PHI from electronic health records to make them more accessible for research. Most de-identification techniques have focused on removing all PHI from text such as discharge notes, radiology or pathology reports, or progress notes (see [9,10] for reviews) in order to allow the text to be shared with collaborators or other researchers. These techniques focus on scrubbing all PHI elements from the medical record data through either lexical, heuristic or pattern based systems or machine learning approaches. However, many collaborative research data sets consist primarily of structured data, which may or may not be allowed to contain PHI elements depending on the IRB agreements. For example, some elements of PHI, such as names or medical record numbers, may need to be removed from the research data sets, while other elements such as birth date and gender may be allowed to support the research. In these cases, protecting PHI during research may primarily require comprehensive review of the research data sets prior to disclosure to ensure that only allowable PHI is included.

Existing PHI review techniques primarily rely on humans to review the data and interpret and apply the PHI restrictions correctly. However, reviewing large, complex data sets can be difficult: study data sets frequently include thousands to millions of records and it’s unrealistic to expect the data reviewer to review every record for data that may qualify as PHI. Methods that reduce the burden on the data reviewer by automatically identifying possible PHI in collaborative data sets have the potential to significantly reduce the probability of accidental PHI disclosure.

In this work, our goal was to create an automated process that would reduce the manual effort of checking the research data sets for PHI. The purpose was not to scrub the data sets of PHI, but to alert the researchers of PHI contained in the data sets for cross-checking against IRB and DUAs. We formed a group of 5 experienced multi-site programmers (PHI Work Group) to identify and address the most common causes of accidental PHI disclosure. The group identified five major problems that may lead to accidental disclosure of PHI:

1. Inclusion of data sets meant to be retained locally in the data that gets transferred to the lead site.

2. Failure to substitute a study-id for patient identifiers.

3. Failure to “scrub” patient identifiers, such as medical record numbers and social security numbers from the data set prior to transfer.

4. Inclusion of dates that indicate rare characteristics, such as advanced age (i.e. age > 89)

5. Indications of small populations with rare disorders.

To address these issues, we developed a macro to identify PHI in SAS data sets prior to data release.

## Methods

### Data environment

The HMORN is a consortium of 19 health care delivery organizations that conducts collaborative research on a wide variety of healthcare topics [2]. To facilitate collaborative research across disparate healthcare delivery organizations, the HMORN has developed a set of standardized data specifications for a virtual data warehouse (VDW) [11]. To obtain data for multi-site research projects, HMORN analysts at the lead research site develop and distribute SAS scripts using common variable names to reference the standardized data structures. Participating sites run the scripts within their own environment and transfer the resulting data sets to the lead site for final analysis. The transfer data sets can range from aggregate counts to patient-level data about encounters, diagnoses and procedures, prescriptions, and lab test results depending on the research needs, the DUA and the IRB agreement.

Transfer of data from participating sites to the lead research site represents the greatest risk of inappropriate PHI disclosure during the research project. Every effort is made to restrict the transferred data to the minimum necessary for complete and accurate study analysis, including de-identification, assigning study IDs, and redacting counts that may identify small populations with rare diagnoses or procedures (referred to as “small cell sizes”). To ensure transfer data sets include appropriate data only, all data sets must be reviewed prior to transfer to ensure that they do not contain PHI beyond what is allowed under data sharing and IRB agreements. Data set review involves identifying and removing any disallowed variables, as well as reviewing the data itself for individual instances of PHI. This is particularly difficult for items such as small cell sizes and ages greater than 89, which can be buried in a single record of a massive data set. Manually checking every data set to make sure it does not contain unauthorized data, including PHI, can be a time consuming and error-prone process.

### Approach

The three project requirements were: 1) the PHI identification process must identify the most common forms of PHI in structured data; 2) the process must be fast to implement to allow quick turnaround; and 3) it must leave the decision about whether PHI is allowable in the hands of research personnel. Based on these requirements, the PHI Workgroup developed an automated program (macro) designed to identify potential PHI in a SAS analytic environment, referred to as the PHI Detection macro. The macro evaluates the directory containing files to be transferred to the lead programming site (transfer directory), identifies SAS data sets, and scans each SAS data set for possible PHI elements. The macro uses a pattern matching approach and is designed to work on data sets that contain structured data. It relies primarily on regular expressions to identify patterns consistent with medical record numbers of social security numbers, field formats to identify dates, and keywords as fieldnames.

The PHI detection macro is designed to run in SAS 9.1 or higher. The macro analyzes the data in the transfer directory at two levels: a high-level overview of all files in the directory for comparison against the programming workplan, and a detailed analysis of the data in each data set. The results of all analyses are printed to a PDF report for review prior to data transfer. The code for the PHI detection macro can be downloaded from https://github.com/HMORN/phi_macros.

The high-level analysis consists of a scan of all files in the transfer directory. The macro creates a summary listing of each file type and the number of files matching that file type in the directory. It also creates a directory listing of each file in the directory, including the name and file type. For SAS data sets, the directory listing also indicates the creation date, modification date and the number of records in the data set. The detailed analysis provides four separate checks on each data set. The first data check looks for variable names that may indicate common PHI elements such as personal identifiers, birth dates, health encounter dates and death dates. Site-specific variable names that may indicate PHI can be specified as a pre-defined global variable. For example, sites which use the variable med_rec_no to refer to a a patient's medical record number may want to ensure that med_rec_no does not occur in transfer data sets. All variable name checks are case insensitive. Table 1 provides examples of some of the strings evaluated in this data check. An example of a site-specific string is shown in the last row of Table 1.

**Table 1:** Example of strings that may indicate PHI if they are used as variable names

**Type of PHI**	**Potential variable names**
Medical Record Number	MRN, Medical_Record_Number, Medical_Record_Num, Medical_Record_No, Med_Rec_No, Med_Record_Num,
Subject Name	Name, First_Name, Last_Name, birth_name, maiden_name, Surname
Social Security Number	SSN, social_security_number, SocialSecuityNumber, socsec
Birth/death dates	Birth_date, bday, birthDate, death_date, deathDay
Health encounter dates	Encounter_dt, enc_dt, encDt
Locally “forbidden” variable names	Medical_history_number|MHN|Pat_ID|Pat_MRN_Id

This list is not exhaustive.

The second data check performed on each transfer data set evaluates strings in the data set to determine if the data matches a regular expression that represents the site’s standard personal identifiers (i.e. Medical Record Numbers). Regular expressions are a highly flexible method for defining text strings to be used in string comparisons. For example, a medical record number that consists of 8 to 9 numeric characters would be defined as “(^\d{8, 9}\s)”, and would match the string “12345678” in the data set. The macro uses the SAS function prxmatch to compare character data in the data set to the regular expression. The macro can be customized to evaluate every record, or to restrict the evaluation to a specific number of records to improve processing speed.

A third data check evaluates the transfer data sets contain dates that may reflect birth, death or healthcare utilization dates. The data check further evaluates the contents of all date variables to determine whether the date may indicate a person over age 89. The macro uses 89 as the default age, as specified in the Privacy Rule [7], but individual sites can override the default to flag younger ages. Variables are considered date variables if either the variable format is a date type, or the variable name contains the word “date.” For each date variable, the macro reviews all records to determine if the data set contains any dates that may indicate the patient was older than 89. The macro also looks for variable names containing the word “age” and looks for records that may identify individual patients who are older than 89.

The purpose of the fourth data check is to identify small groups that may indicate rare conditions or treatments. According to the privacy rule [7], individuals with rare or uncommon diagnoses or conditions may be identifiable even when the 18 specific patient identifiers are removed [7] and therefore information about individuals with such rare conditions should be considered PHI. The macro scans all numerical variables in the transfer data sets to identify values between 1 and 5 (inclusive) and prints a report listing all numerical variables with values between 1 and 5.

The final step in the detail analysis is to print 5 sample records to the PHI report. The sample records allow the data reviewer to manually review all variable names and a subset of data to find any potential PHI the automated analysis may have missed.

### Evaluation

We measured the performance of the phi detection macro by testing it against two types of data. The first test data set consisted of fake data that contained multiple examples of PHI: dates, names, addresses, medical record numbers, birth dates, and social security numbers. We ran the macro on the directory containing the fake PHI data and evaluated how many of the PHI types the macro identified.

For the second test of the PHI macro, we created a collection of 100 data sets from previous research projects. The collection included data sets that were shared with other research sites, as well as data sets that were meant to be retained locally. Data sets in the collection contained a variable level of PHI: some data sets contained names, addresses and medical record numbers, while other data sets contained no PHI. We restricted each data set to 50 records to facilitate manual review.

We used manual review as our gold standard of PHI detection. Every data set was reviewed by two people to ensure that all examples of PHI were identified. For each instance of PHI, we captured the field name and type of PHI to a log. Once all test data sets had been hand reviewed, we executed the macro on the directory containing the collection of data sets, and cross-checked the PHI report against the PHI log generated from the hand review process.

## Results

The HMORN PHI Workgroup developed a SAS macro designed to identify PHI in research data sets. The PHI detection macro scans the transfer directory to identify files to review and performs a detailed analysis of each SAS data set in the transfer directory. The macro produces a PDF report that the site data reviewer, usually the research analyst or project manager, can review to ensure that any data to be released from the collaborating site is consistent with the data sharing and IRB agreements and does not contain unauthorized PHI. Examples of complete reports using fake patient data can be found at http://mapri.kaiserpermanente.org/research/mapri-sample-reports/.

**Figure 1:**
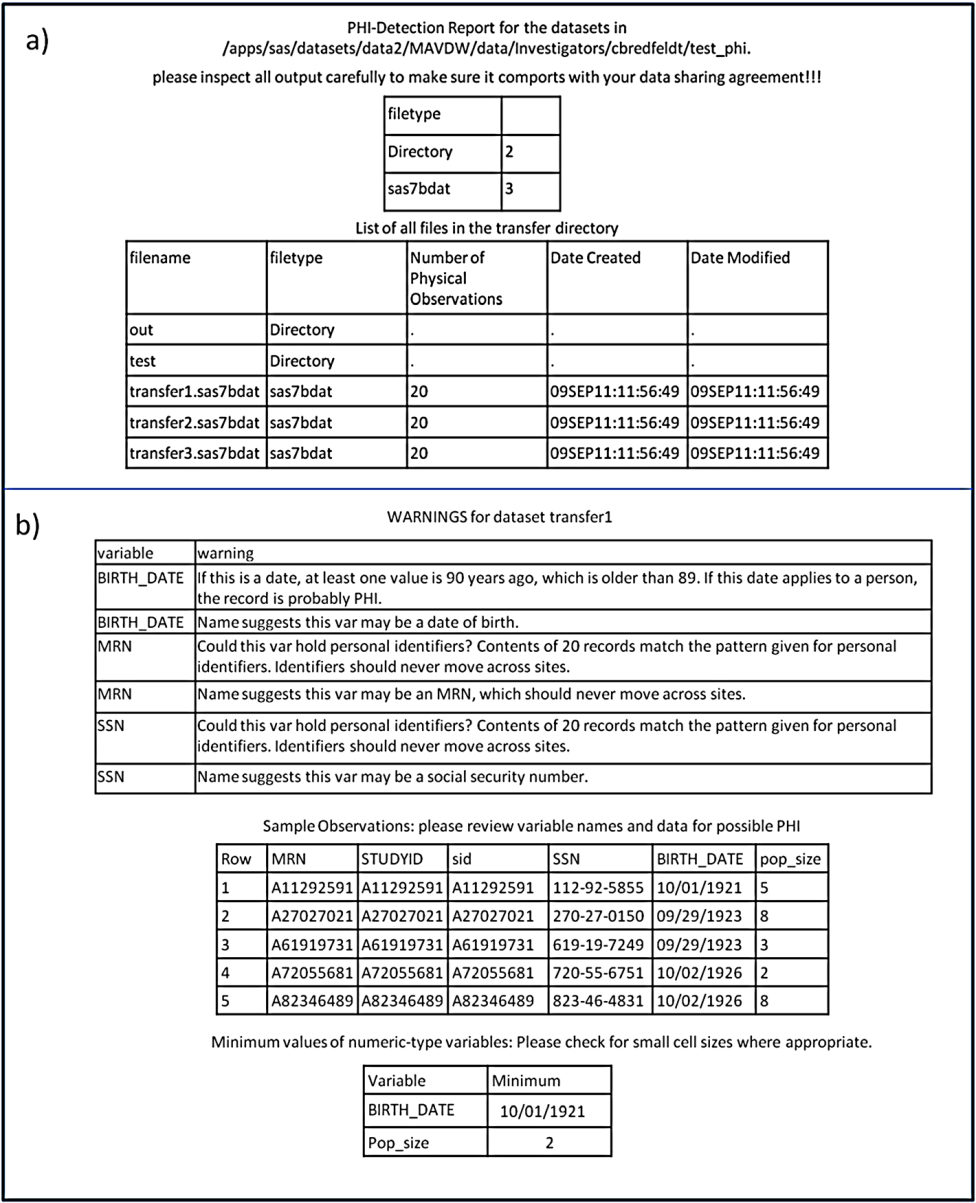
**Example of a report from the PHI detection macro.** (**a**) High level analysis showing the number of files in the transfer directory, by file type. (**b**) Detailed report of potential PHI in the sas data set files in the transfer directory (top), as well as example records (middle) and minimum values of numerical variables.

The PHI detection report contains two sections: the overview and the detail section. Figure 1 shows an example of the PHI detection report. The overview section provides a count of files by file type and a listing of all files in the transfer directory. Figure 1a illustrates a typical example of the overview section of the PHI Detection report. The file listing includes a record count for all SAS data sets, as well as the date each data set was created and modified. Both the file count and the file listing can be compared to the expected output described in the program’s workplan to evaluate whether the program has produced the correct data sets and to ensure there are no unexpected files in the transfer directory. In addition, when the data sets contain individual-level data, the record count for population data sets can be compared to the estimated size of the target population to ensure that the program is identifying the appropriate population.

The detail section of the PHI detection report (example shown in Figure 1b) contains information about each data set in the transfer directory. The report contains three subsections for each data set: warnings regarding potential PHI the program has identified, sample records, and information about low values of the numeric variables. The warnings subsection contains the results of all automatic PHI checks, including checks for “forbidden” variable names, comparisons of string data to potential study identifier formats, and checks for dates greater than the cutoff value. If any of these checks identify data that may be PHI, a warning is printed to the report describing the potential problem. An example of these warnings is shown in the top section of Figure 1b.

The second subsection of the detailed analysis of each data set displays a small number of example records from the data set for manual inspection. In cases where potential problems have been identified such as variables that meet the defined pattern for a medical record number, or records with small or zero numerical values, records containing the identified concerns are selected for review. In cases where the macro has not identified any specific concerns, five random records are selected for review. Figure 1b shows the case where the macro has identified two potential concerns: records with data that contain patterns that are consistent with PHI, and records with numerical values that may indicate critically small populations. The sample records allow the data reviewer to examine specific examples to determine if the records genuinely contain PHI, and prompt the reviewer to check any PHI found in the data set against the IRB and data sharing agreements.

The final subsection identifies the minimum value of all numeric variables in the data set. This analysis assumes that numeric variables may represent counts of events or members of a population. If the minimum value of a count variable is zero, it may represent missing data and indicate that there is an error in the code that needs to be reviewed. In addition, according to HIPAA, populations with between 1 and 5 people may be considered PHI if they have rare conditions because it is relatively easy to identify the individuals in the population. Thus, if the minimum value of a numeric variable is between 1 and 5 (inclusive), that variable should be inspected to ensure that either it does not represent a population count, or low population counts are allowable in the transfer data sets based on the IRB approval and data sharing agreements.

Performance: We first evaluated the PHI Detection macro on three test data sets containing a variety of PHI, including medical record numbers (MRNs), social security numbers, ages greater than 89, birth dates, and small populations. The PHI report correctly listed all 20 fields that either contained PHI or were likely to contain PHI based on the field name (one field labeled “MRN” contained study-specific identifiers, and was flagged by the report).

We next evaluated the PHI Detection macro on 100 research data sets, comparing the PHI detected by the macro to that found on manual review. The test data sets were drawn from a combination of site-specific data (likely to contain PHI) and collaborative data (should not contain extensive PHI). Each test data set was restricted to the top 50 records to improve accuracy of the manual review process. Manual review was performed by two experienced research analysts to ensure accuracy. The PHI Detection macro correctly identified 111 out of 113 instances of PHI in the test data sets, for a recall of 0.98. There were also 26 false positives out of a total of 809 data fields, for a specificity of 0.96. The macro has a precision of 0.81, and an F-score of 0.88.

## Discussion

We developed a SAS macro program to identify potential PHI in collaborative data sets. The macro scans all data sets in a given directory for variable names, data patterns, and numeric values that may represent PHI. Metadata about the data sets in the directory is printed to a PDF report, along with any warnings identified in the data. The analyst or investigator can then use the report to determine if the research data sets contain unauthorized PHI.

Managing PHI carefully in multi-site research environments is critical to protecting our patients and complying with federal laws. In this paper we describe an approach for identifying PHI in collaborative research environments that work primarily with structured data, such as utilization records, lab data and patient vitals. Our approach uses a relatively simple pattern matching method that leverages the metadata contained in structured data fields through two techniques: 1) pattern matching on field names; and 2) using field data types to identify dates. We further use regular expressions to find fields containing distinctive identifiers such as medical record numbers and social security numbers. By leveraging the structure of the data sets, we are able to use a relatively small dictionary of 21 terms that can be customized as needed for each site. Despite the limited data dictionary, the macro has high sensitivity and specificity in identifying common PHI elements such as medical record numbers, dates and patient names.

The majority of previous efforts at PHI protection for multi-site research have focused on de-identifying or anonymizing free-text documents such as pathology reports and progress notes (see [9,10]). Free text can contain complex forms of PHI such as proper names, making them much more difficult to scrub. These efforts have led to the development of more sophisticated machine learning and lexical, heuristic and pattern based methods for identifying PHI. These methods often require a large corpus to train the machine learning algorithms, or an extensive dictionary to support the pattern matching approach [9]. In cases where multi-site research is conducted entirely on structured data where there is less variability in the data and a more limited set of potential PHI elements, these approaches may be more sophisticated and resource intensive than is necessary. A simple method that quickly and accurately scans a large body of data to produce a PHI report could significantly improve the probability of identifying unauthorized PHI prior to transfer.

Our approach differs from many PHI-protection approaches in that it focuses on identifying, but not removing, potential PHI. Previous privacy protection methods on structured data have focused on anonymizing the data by removing data points until individuals are statistically indistinguishable [12]. However, this method is open to reverse engineering and may remove critical data attributes [13]. In this work, we start from the perspective that many multi-site research projects have IRB approval to share certain types of PHI necessary for adequately answering the research question. The aim with this work was to generate a report that would allow a human reviewer to determine whether any PHI identified in the research data sets can be shared with other research sites based on both IRB and DUAs.

The macro is not meant to replace human review of the data sets. Rather, it is intended to increase the efficiency and completeness of the data review. Manual review can be tedious and inaccurate for research projects containing multiple data sets with thousands to millions of records each. By explicitly generating warnings regarding potential PHI elements in the data sets, the macro draws attention to data that may need closer review prior to release. In addition, the PHI detection macro is able to review every record for inappropriate dates, ages or counts, which can be impossible for a human to do in a large, complex data set. Finally, by providing a clear list of all files in the transfer directory, the macro makes it easier to confirm that only the intended files are included in the transfer directory.

As with other PHI-protection procedures, the macros are only effective if multi-site programmers use them properly. The four most common problems that may reduce the effectiveness of the macros is specifying the wrong directory for data review, failing to adequately specify the regular expressions used to define key identifiers, failing to review the reports produced by the macros, and failing to run the macros prior to transferring the data. Three of these four problems can be addressed by using a PHI checklist for final review. Such a checklist would prompt the data reviewer to compare the information in the PHI report to the information in the program’s workplan to ensure the transfer directory contains the right data and any PHI identified in the report is allowable under the terms of the IRB approval and DUAs. The fourth problem (incorrect specification of the regular expressions) can be addressed by testing the regular expression definition against some sample data using publicly available tools such as http://www.regextester.com/.

## Conclusions

The PHI Protection macros described in this paper are intended to reduce accidental PHI disclosure in multi-site research using structured data by automating the review of shared data sets. The automated review provides a report describing all files in the transfer directory, including warnings if it finds common indicators of PHI in either the data sets or the programming logs. When used in conjunction with a careful, checklist-guided manual review of the data for unauthorized PHI, the macro has the potential to significantly reduce accidental PHI disclosures.

## Abbreviations

PHI: Protected health information; IRB: Institutional review board; DUA: Data use agreement; HIPAA: Health insurance portability and accountability act.

## Competing interests

The authors declare that they have no competing interests.

## Authors’ contributions

CB: Designed evaluation phase and drafted the manuscipt. SP: Conceived of the study and led study design. AB: Contributed to study design and evaluation phase. RP: Led macro design. PH: Contributed to study design and evaluation phases. All authors read and approved the final manuscript.

## Pre-publication history

The pre-publication history for this paper can be accessed here:

http://www.biomedcentral.com/1472-6947/13/39/prepub
